# Supplementary material for: Hot Electron Extraction in SWCNT/TiO2 for Photocatalytic H2 Evolution from Water
Source: Nanomaterials (Basel). 2022 Oct 29;12(21):3826. doi: 10.3390/nano12213826 (PMC9654061; doi:10.3390/nano12213826)
Supplement: Supplementary file 1 [file nanomaterials-12-03826-s001.zip › nanomaterials-1983434-supplementary.pdf]

Supplementary Information

Hot Electron Extraction in SWCNT/TiO_2 for Photocatalytic H_2 Evolution from Water

Masahiro Yamagami [1], Tomoyuki Tajima [1], Zihao Zhang [2], Jun Kano [2], Ki-ichi Yashima [3], Takana Matsubayashi [3], Huyen Khanh Nguyen [3], Naoto Nishiyama [3], Tomoya Hayashi [3] and Yutaka Takaguchi [3,*]

[1] Graduate School of Environmental and Life Science, Okayama University, 3-1-1 Tsushima-Naka, Kita-ku, Okayama 700-8530, Japan

[2] Graduate School of Natural Science and Technology, Okayama University, 3-1-1 Tsushima-naka, Kita-ku, Okayama 700-8530, Japan

[3] Department of Material Design and Engineering, Faculty of Sustainable Design, University of Toyama, Toyama 930-8555, Japan

* Correspondence: tak@sus.u-toyama.ac.jp; Tel.: +81-(0)76-445-6837

1. Apparatus used in a photocatalytic hydrogen evolution reaction

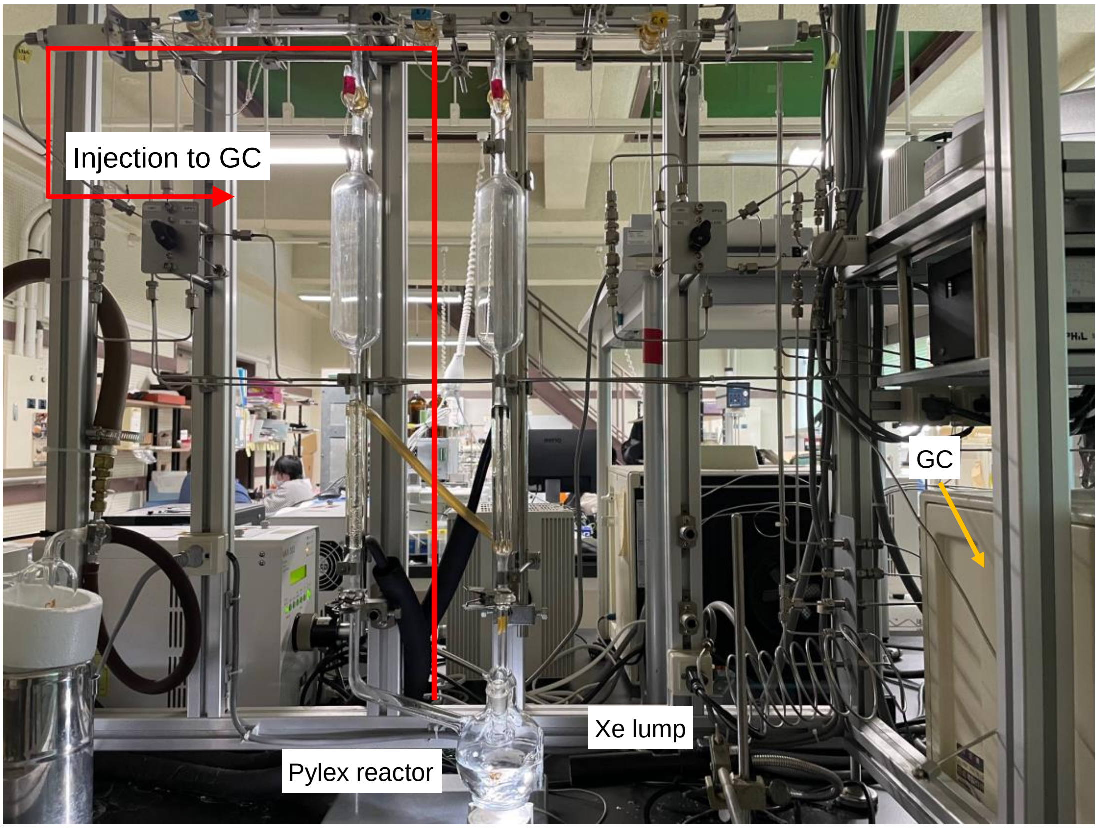

Figure S1 Photograph of closed circulation system used in a photocatalytic hydrogen evolution reaction.

2. Absorption spectra before/after the hybridization of SWCNTs with TiO_2

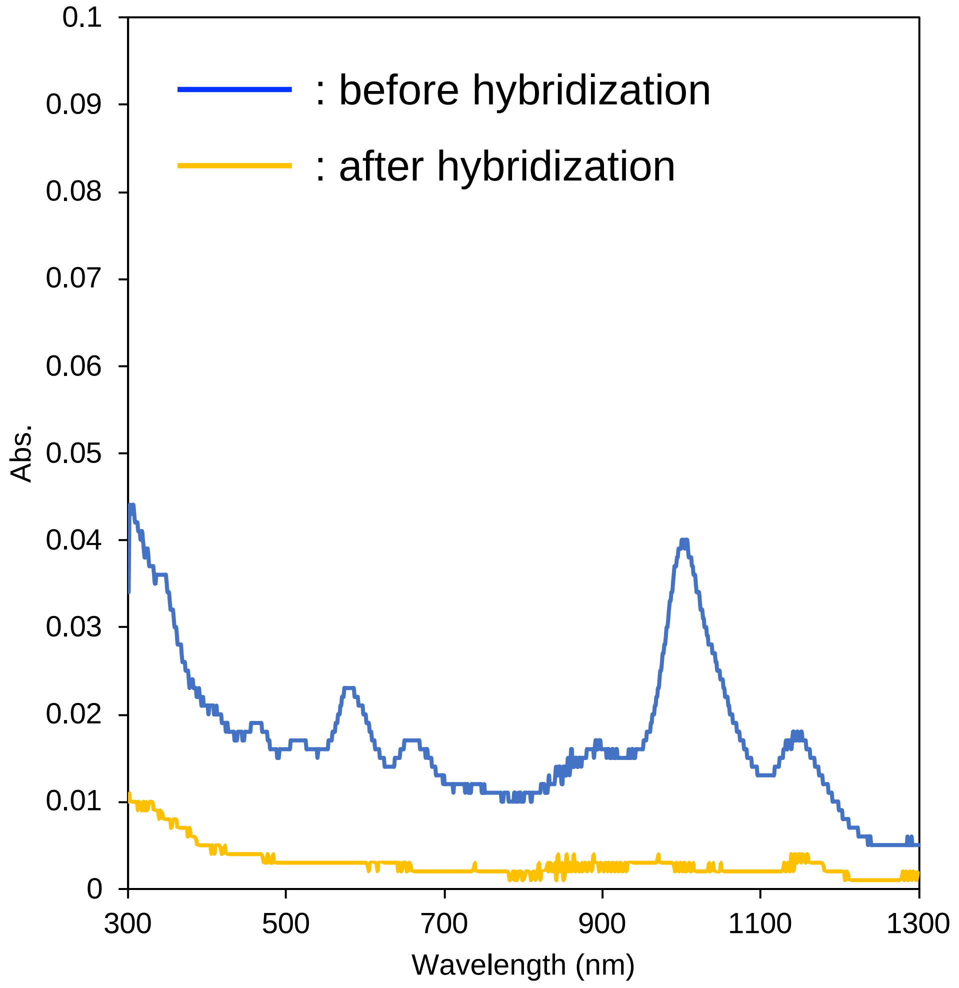

Figure S2 Absorption spectra of supernatant before/after the hybridization of SWCNTs with TiO_2.

3. **HR-SEM images of SWCNT/TiO_2/Pt**

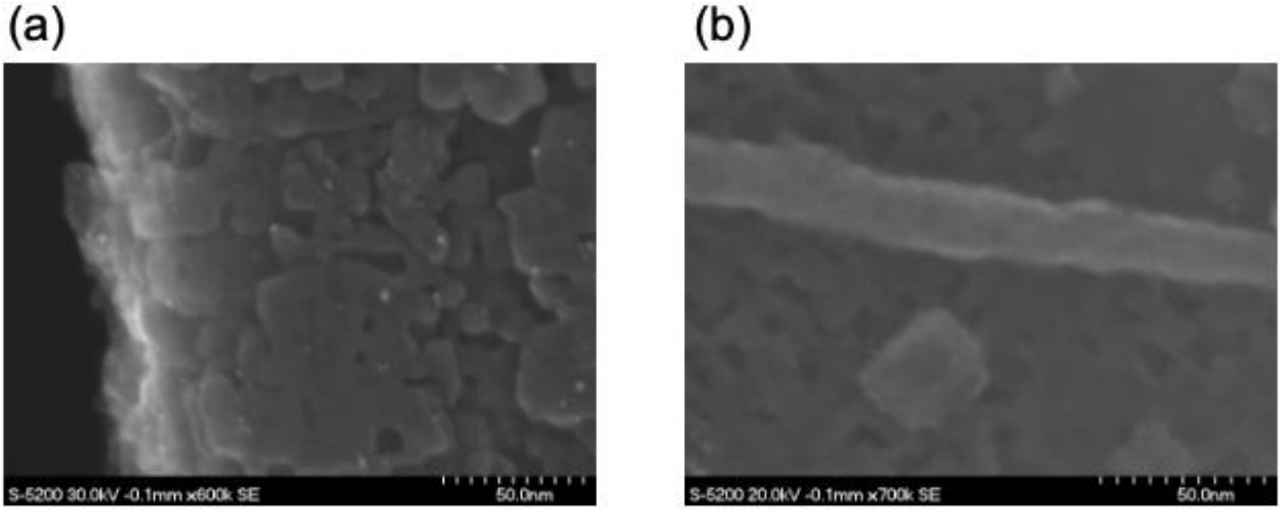

Figure S3 HR-SEM images of SWCNT/TiO_2/Pt using in-lens detector.

4. 2D excitation/emission spectra before and after the hybridization of SWCNTs and TiO_2

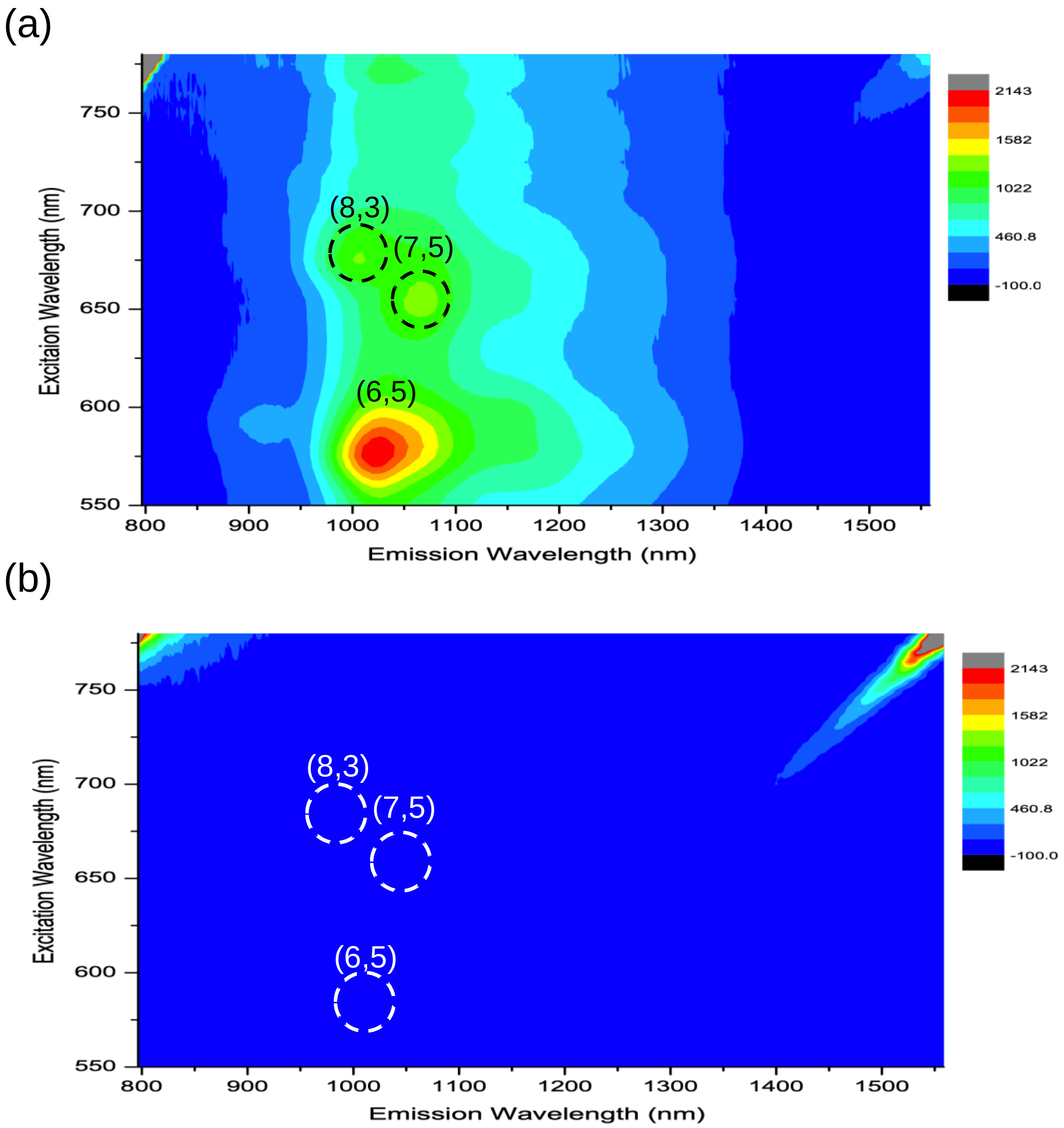

Figure S4 Two-dimensional PL intensity maps of (a) SWCNT/BDD-dendrimer(COOH) nanohybrids and (b) SWCNT/TiO_2/Pt

5. **A time course of the hydrogen evolution reaction using SWCNT/TiO_2/Pt.**

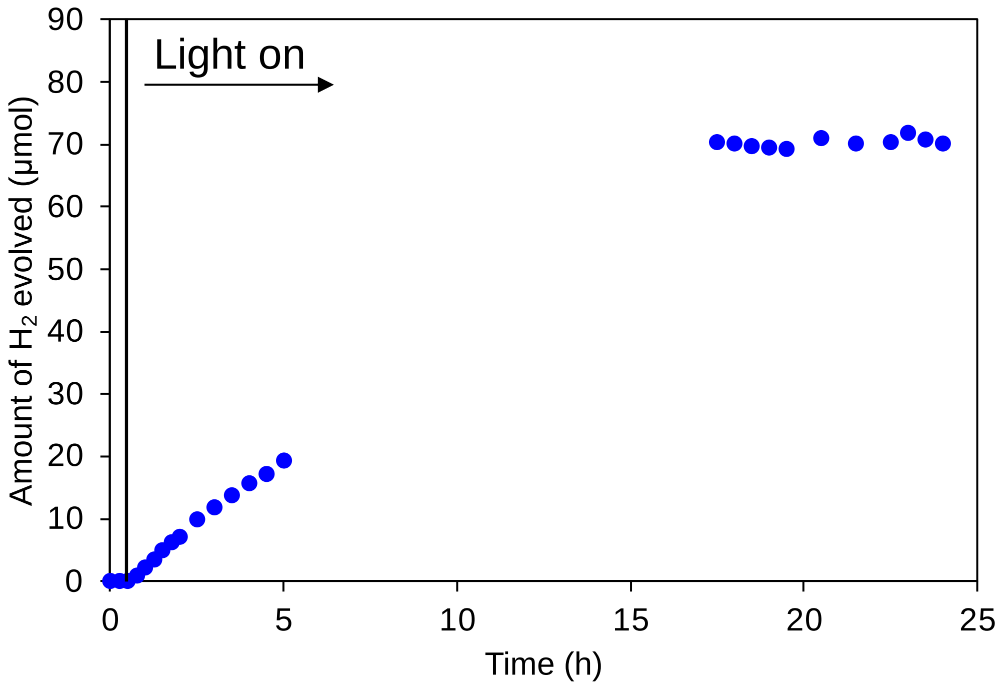

Figure S5 A time course of photocatalytic hydrogen evolution using SWCNT/TiO_2/Pt under visible light irradiation ($\lambda > 422$ nm).

6. Photocatalytic hydrogen evolution using SWCNT/TiO_2(P25)/Pt

The TiO_2 Aeroxide P25 (anatase : rutile = 8:2) was purchased from NIPPON AEROSIL CO., LTD. supplied by Evonik, Germany. P25/Pt particles were prepared by the same method as TiO_2/Pt. 10 mg of 1 wt% Pt-loaded P25 and 125 μL of SWCNT/BDD-dendrimer(COOH) nanohybrids were added in 10 mL of water. Then, stirring for 30 minutes and immersed for overnight in the dark. After that, the supernatant was removed by decantation, the sample was dried and kept in the dark (denoted as SWCNT/TiO_2(P25)/Pt). Figure S6 shows a time course of photocatalytic hydrogen evolution reaction over SWCNT/TiO_2(P25)/Pt under visible light irradiation (λ > 422 nm). The hydrogen production rate of 7.3 μmol/h was observed without any induction period.

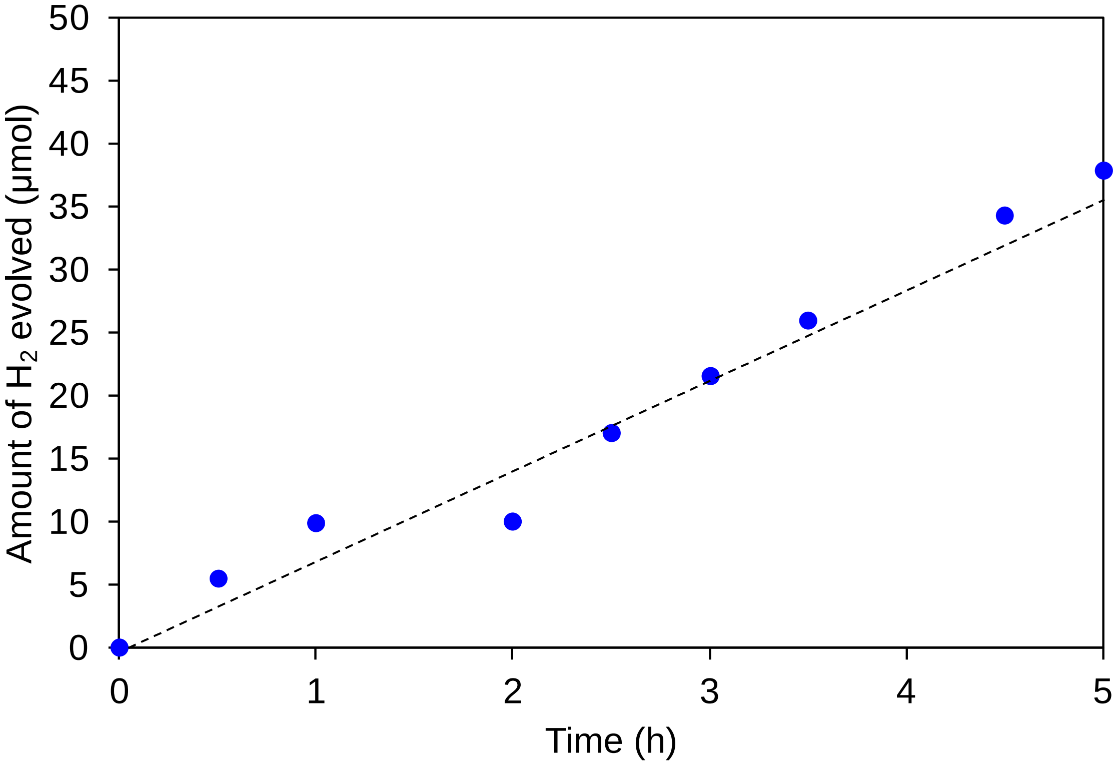

Figure S6 A time course of H_2 evolution from water over SWCNT/TiO_2(P25)/Pt under visible light irradiation (λ > 422 nm).
